# Postbiotics and Nicotinamide Utilize Distinct Mechanisms to Improve Skin Barrier Integrity, Inflammation, and Keratinocyte Differentiation

**DOI:** 10.1111/all.70225

**Published:** 2026-01-23

**Authors:** Yagiz Pat, Duygu Yazici, Huseyn Babayev, Sena Ardicli, Xiangting Bu, Sheri Simmons, Anthony Almada, Christine Avena, Tye Jensen, Raja Dhir, Patrick Westermann, Asuncion Garcia‐Sanchez, Manru Li, Ozge Ardicli, Can Zeyneloglu, Marco Pane, Angela Amoruso, Christoph Messner, Ismail Ogulur, Yasutaka Mitamura, Mubeccel Akdis, Cezmi A. Akdis

**Affiliations:** ^1^ Swiss Institute of Allergy and Asthma Research (SIAF) University of Zurich Davos Switzerland; ^2^ Department of Genetics, Faculty of Veterinary Medicine Bursa Uludag University Bursa Türkiye; ^3^ SEED Inc. Co. Los Angeles California USA; ^4^ Precision Proteomics Center, Swiss Institute of Allergy and Asthma Research (SIAF) University of Zurich Davos Switzerland; ^5^ Department of Biomedical and Diagnostic Sciences University of Salamanca Salamanca Spain; ^6^ Division of Food Processing, Milk and Dairy Products Technology Program, Karacabey Vocational School Bursa Uludag University Bursa Türkiye; ^7^ Probiotical Research S.r.l. Novara Italy

**Keywords:** epithelial barrier, ex vivo human skin, inflammation, postbiotics, transcriptomics

## Abstract

The modulation of immune responses and tissue regeneration by postbiotics is a rapidly advancing area in skin care. Here, we show that whole‐cell postbiotics derived from *Bifidobacterium breve* , *Limosilactobacillus reuteri*, and *Ligilactobacillus salivarius*, along with nicotinamide (NAM), enhance keratinocyte growth, differentiation, and skin epithelial barrier integrity in ex vivo human skin, as determined by electrical impedance spectroscopy (EIS), multiomics, and machine learning. *B. breve* promoted keratinocyte differentiation and suppressed inflammatory pathways, while *L. reuteri* and *L. salivarius* primarily reduced inflammatory pathways. Although NAM downregulated keratinocyte differentiation, it exerted anti‐inflammatory effects. Machine learning analyses linked EIS changes to certain genes, highlighting strain‐specific mechanisms. In addition, *B. breve* , *L. reuteri* , and NAM mitigated a common skin cleanser‐induced skin epithelial damage, further supporting their therapeutic potential. In conclusion, integrating skin barrier measurements with omics and machine learning enabled the dissection of essential anti‐inflammatory and keratinocyte differentiation mechanisms and genes of a strengthened skin barrier.

## Graphical abstract

Integrating skin barrier measurements with omics and machine learning, we found that postbiotics and NAM regulate skin homeostasis through distinct, strain‐ and dose‐dependent mechanisms. Postbiotics primarily modulate skin barrier function by promoting keratinocyte differentiation and suppressing inflammation. Together, integrated functional, molecular, and machine learning analyses identify key pathways underlying skin barrier regulation. EIS, electrical impedance spectroscopy; ETV4, ETS translocation variant 4; KRT81, keratin 81; LATS2, large tumor suppressor kinase 2; LC–MS, liquid chromatography‐mass spectrometry; mM, millimolar; NAM, nicotinamide; NOTCH1, neurogenic locus notch homolog protein 1; OVOL2, ovo‐like zinc finger 2; RNA‐seq, RNA sequencing; w/v, weight/volume.

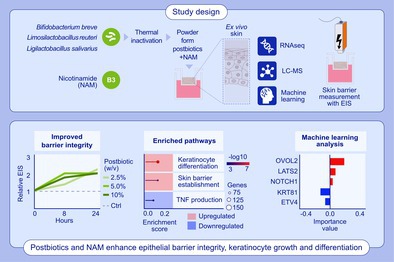

## 1 Introduction

The skin epithelial barrier is one of the first lines of defense against environmental factors such as pathogens, allergens, and harmful substances [1]. The epidermis, the outer protective layer composed of stratified squamous epithelium, sits above the dermis, which contains connective tissue, blood vessels, glands, and resident immune cells [2]. Keratinocytes within the epidermis undergo a highly regulated differentiation program, culminating in the formation of the cornified envelope, a specialized protein‐ and lipid‐rich structure that provides mechanical strength and barrier function. This transformation strengthens the mechanical and barrier functions of keratinocytes as they transition into corneocytes, forming the stratum corneum. The cornified envelope, enriched with proteins such as late cornified envelope (LCE) family members, involucrin (IVL), and filaggrin (FLG), provides mechanical and chemical resilience [3]. Disruptions in the skin epithelial barrier, whether due to genetic, environmental, or inflammatory factors, can lead to skin disorders such as atopic dermatitis, rosacea, alopecia, and psoriasis, highlighting the importance of skin homeostasis and barrier integrity [1, 4].

In recent years, accumulating evidence has demonstrated the relationship between the skin microbiome and epidermal barrier function [5]. The use of probiotics for skin health dates back to 1912 for treating acne, followed by numerous studies that have explored their dermatological benefits [6, 7, 8, 9]. However, the interactions between bacteria and skin homeostasis extend beyond live microorganisms. The concept of “postbiotics,” broadly defined as inanimate microorganisms and/or their components that confer health benefits, has gained increasing attention in skincare and supplements as a way to modulate inflammation, support tissue repair, and enhance barrier function without requiring the administration of live bacterial cells [10, 11]. Compared to conventional probiotics, postbiotics lack the potential risks related to live bacteria administration, making them ideal candidates for dermatological applications [10, 12]. Studies have demonstrated that postbiotic fractions, ranging from short‐chain fatty acids to peptides and bacteriocins, can promote antimicrobial defenses, mitigate inflammatory signaling, and regulate keratinocyte proliferation [13, 14]. Among the most well‐studied postbiotic‐producing genera are *Bifidobacterium* and *Lactobacillaceae*, which exhibit immunomodulatory properties and epithelial barrier‐enhancing effects [6, 7, 8, 9, 15, 16].

Additionally, nicotinamide (NAM), a water‐soluble form of vitamin B3, is gaining recognition as a small molecule with robust effects on the skin barrier. NAM has been shown to stimulate ceramide biosynthesis, enhance keratinocyte proliferation, and reduce transepidermal water loss [17, 18]. Although NAM exerts overlapping benefits with certain postbiotic compounds, its mode of action involves direct effects on oxidative stress pathways and inflammatory cascades [19, 20].

In the present study, we investigated the effects of postbiotic strains, *Bifidobacterium breve* ( *B. breve* ), *Limosilactobacillus reuteri* ( *L. reuteri* ), and *Ligilactobacillus salivarius* ( *L. salivarius* ), on skin barrier integrity and inflammatory responses using an ex vivo human skin model. Electrical impedance spectroscopy (EIS), a noninvasive, rapid method previously validated in skin research, was used to monitor changes in skin barrier function in real‐time [21]. We also conducted comprehensive transcriptomic analyses to map gene expression profiles associated with keratinocyte differentiation and inflammation‐related pathways. In addition, machine learning analysis revealed that postbiotics improve and maintain the skin epithelial barrier by regulating distinct cellular pathways and genes, paving the way for discovering new biomarkers for treating or improving the skin epithelial barrier integrity. Furthermore, we showed the beneficial effects of postbiotics and NAM in a functional manner by conducting a mitigation study against a commonly used skin cleanser surfactant. Our results indicate a dose‐, strain‐, and time‐dependent effect of *B. breve* , *L. reuteri* , and *L. salivarius* on bolstering epithelial barrier function while modulating inflammatory mediators. A comparison with NAM provided a basis for distinguishing postbiotic‐driven mechanisms from those arising via known modulators of keratinocyte function. Specifically, we highlight the different thresholds at which these postbiotics transition from protective to potentially inflammatory, emphasizing the importance of dose optimization in the formulation of topical preparations, including postbiotics.

## 2 Results

### 2.1 *B. breve* and *L. reuteri* Postbiotics Increase Skin Epithelial Barrier Integrity

**FIGURE 1:**
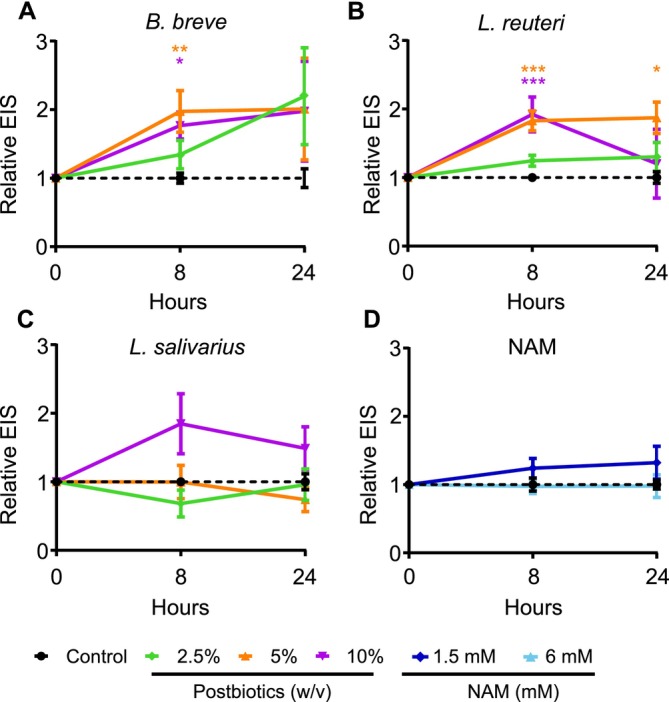
Postbiotics increase the EIS measurement in ex vivo skin tissues. (A) *B. breve* ; (B) *L. reuteri* ; (C) *L. salivarius*; (D) NAM. Postbiotics (2.5%, 5%, and 10% w/v) and nicotinamide (NAM; 1 and 6 mM) were applied apically to ex vivo human skin tissues. Electrical impedance spectroscopy (EIS) was measured at baseline, 8, and 24 h. For the experiments, at least two different donors with 2–3 technical replicates were used. In total, eight donors were used. *B. breve* , *Bifidobacterium breve* ; EIS, electrical impedance spectroscopy; *L. reuteri* , *Limosilactobacillus reuteri*; *L. salivarius* , *Ligilactobacillus salivarius*. *p < 0.05, **p < 0.01, ***p < 0.001.

To assess the effect of postbiotics and NAM on skin epithelial barrier integrity, we measured the EIS of ex vivo skin samples obtained from healthy individuals following the treatment with 2.5%, 5%, and 10% (w/v) [2.5 × 10^7^, 5 × 10^7^, and 10^8^ TFU/mL (total forming units/mL), respectively]doses of postbiotics and 1.5 and 6 mM NAM (Figure 1). *B. breve and L. reuteri* increased the EIS values after 8 h at 5% and 10% doses (Figure 1A). Although the EIS at 24 h decreased slightly from its 8‐h peak, the barrier integrity remained above baseline for the 5% concentration (Figure 1B). *L. salivarius* and NAM showed increasing EIS trends, which were not statistically significant (Figure 1C,D). Overall, these data demonstrated that postbiotic treatment causes species, time, and dose‐dependent increases in skin epithelial barrier integrity. The investigation of substantial numbers of ex vivo skin tissues in time kinetics demonstrated robust skin barrier data up to 24 h, showing consistency in replications.

### 2.2 Postbiotics Modulate Keratinization, Inflammation, Metabolism, Cellular Stress, and Cell Death Pathways in the Skin

**FIGURE 2:**
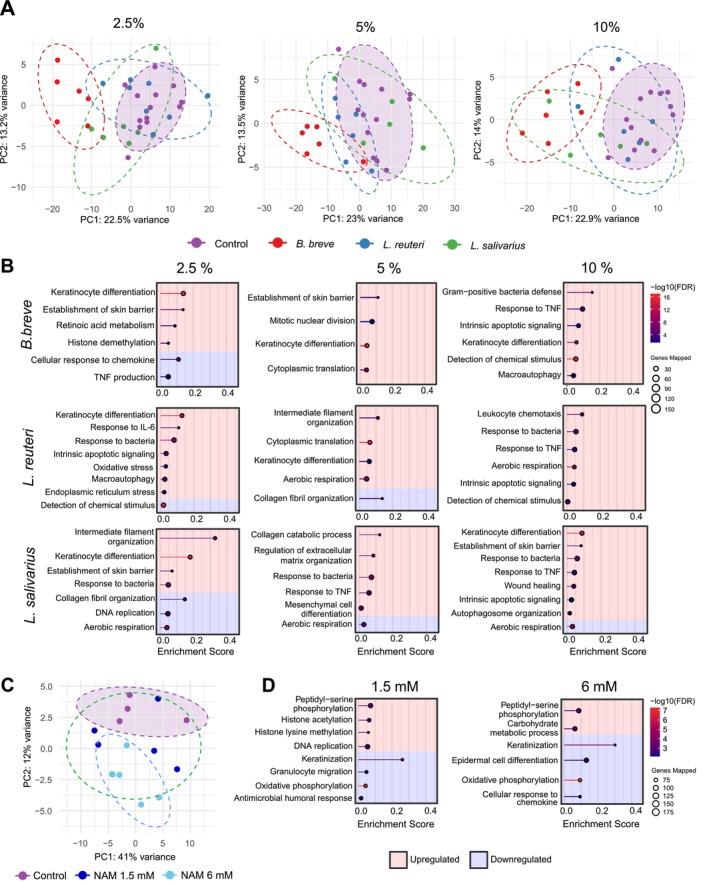
Transcriptomic analysis of postbiotics in ex vivo skin tissues. (A) PCA plots of postbiotics with doses of 2.5%, 5% and 10% (w/v). GSEA analysis of (B) *B. breve* , *L. reuteri* , *L. salivarius* in ex vivo skin tissues with doses of 2.5%, 5% and 10% (w/v). (C) PCA plot of NAM with doses of 1.5 mM and 6 mM. (D) GSEA analysis of NAM. The red background represents the upregulated while the blue background represents the downregulated pathways. The number beside the pathways shows the significant genes belonging to the indicated pathway. RNA sequencing was performed with at least two donors with three technical replicates. *B. breve* , *Bifidobacterium breve* ; GSEA, gene set enrichment analysis; IL‐6, interleukin 6; *L. reuteri* , *Limosilactobacillus reuteri*; *L. salivarius* , *Ligilactobacillus salivarius*; NAM, nicotinamide; PCA, principal component analysis; TNF, tumor necrosis factor.

To analyze the mechanism of postbiotic and NAM‐induced skin epithelial barrier increase, we performed RNA‐seq analysis of the ex vivo human skin at 24 h of treatment along with an untreated control. Principal component analysis (PCA) showed that the postbiotics led to a significant separation from the control, indicating a dose‐ and strain‐dependent impact on the transcriptome (Figure 2A). The comparison of up‐ or downregulated pathways by three postbiotics is shown in Figure 2B. Treatment with *B. breve* led to the enrichment of pathways related to skin barrier integrity and immune modulation. At lower concentrations, 2.5% and 5%, the most prominent processes included keratinocyte differentiation, the establishment of the skin barrier, and retinoic acid metabolism with high enrichment scores. Additionally, 2.5% *B. breve* downregulated cellular response to chemokine and TNF production. However, in 10%, inflammatory pathways such as gram‐positive bacteria defense and response to TNF are upregulated. Notably, 10% *B. breve* induced macroautophagy implying activation of inflammation‐related pathways and cell death. Meanwhile, the effect on keratinocyte differentiation continued at 10%. These data show a dose‐dependent regulation of inflammation and cell death‐related pathways.

2.5% and 5% doses of *L. reuteri* upregulated keratinocyte differentiation pathways, but this effect was absent at 10% (Figure 2B). All doses of *L. reuteri* upregulated inflammation and cellular stress responses. Specifically, at 2.5%, *L. reuteri* notably upregulated the response to IL‐6 and oxidative stress, pointing to an impact on pathways that balance inflammation and cellular stress. At 10%, the transcriptomic analysis revealed enrichment in leukocyte chemotaxis and response to bacteria, indicating that higher doses may further increase the recruitment of immune cells, antimicrobial functions, and the regulation of cell death pathways.

Additionally, *L. salivarius* influenced pathways related to tissue remodeling and skin barrier reinforcement (Figure 2B). At 2.5%, pathways such as intermediate filament organization, establishment of skin barrier, and keratinocyte differentiation were upregulated. *L. salivarius* strengthened the cytoskeletal and barrier properties of the skin. However, a downregulation in collagen fibril organization was detected. In addition, at the 5% dose, collagen catabolic process, regulation of extracellular matrix organization, and mesenchymal cell differentiation pathways were upregulated. The data showed that 2.5% and 5% negatively impacted healthy tissue architecture maintenance. At 10%, *L. salivarius* continued to drive response to TNF and response to bacteria, suggesting broader roles in inducing inflammation and cell death. In contrast to the effects of *L. reuteri* , *L. salivarius* led to a decrease in aerobic respiration at all concentrations. This suggests that postbiotics may induce a strain‐dependent metabolic reprogramming effect on skin tissue.

NAM had a distinct effect on the transcriptome compared to postbiotics (Figure 2C,D). 1.5 mM NAM upregulated epigenetic regulation, including histone acetylation, histone lysine methylation, and post‐translational protein modification, such as peptidyl–serine phosphorylation. In addition, it upregulated DNA replication. In contrast to postbiotics, 1.5 and 6 mM NAM downregulated keratinization. In addition, both concentrations downregulated inflammation‐related pathways, including granulocyte migration, antimicrobial humoral response, and cellular response to chemokine. Additionally, its strong antioxidant properties appeared to downregulate oxidative phosphorylation genes.

Overall, these data clearly show strain and dose‐dependent modulation of the transcriptome with different postbiotic strains. The common effects of postbiotics were keratinocyte differentiation, response to bacteria, and innate immune response.

### 2.3 *B. breve* and *L. salivarius* Upregulate Keratinocyte Differentiation Genes in Ex Vivo Skin Tissue

**FIGURE 3:**
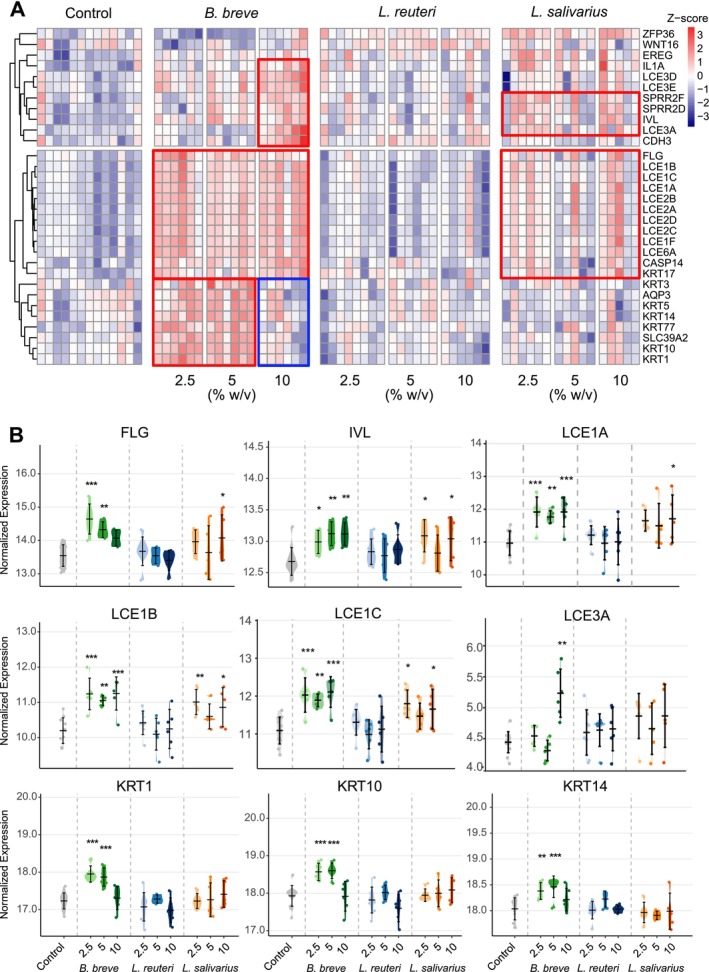
*B. breve* and *L. salivarius* upregulate keratinization in ex vivo skin tissues. (A) The heatmap shows the significant genes related to keratinocyte differentiation (GO:0030216) upon postbiotic treatment for 24 h compared to the control (*p* < 0.05). (B) The violin plots of significant genes related to keratinocyte differentiation (GO:0030216). The data is shown as normalized expression values. *B. breve* , *Bifidobacterium breve* ; *L. reuteri* , *Limosilactobacillus reuteri*; *L. salivarius: Ligilactobacillus salivarius*. *p < 0.05, **p < 0.01, ***p < 0.001.

Transcriptomic analyses revealed that postbiotic application strongly influences the skin epithelial barrier establishment process by upregulating pathways related to keratinocyte differentiation. These findings represent the establishment of the skin barrier and intermediate filament organization, which refers to keratin expression and organization inside the cell. Figure 3A presents the differentially regulated genes associated with the keratinocyte differentiation pathway upon postbiotic application for 24 h. Among the tested strains, *B. breve* exhibited the strongest effect, *L. salivarius* had a moderate effect, while *L. reuteri* showed minimal regulation of keratinocyte differentiation genes. Treatment with *B. breve* resulted in an increase in the expression of genes belonging to the LCE genes, as well as IVL and FLG. These genes are crucial in skin barrier formation by contributing to the formation of the cornified envelope, enhancing epidermal integrity, and protecting against environmental insults. Dysregulation of these genes has been linked to skin disorders such as atopic dermatitis, psoriasis, ichthyosis vulgaris, Vohwinkel syndrome, and progressive symmetric erythrokeratodermia [3, 22, 23, 24, 25].

Both *B. breve* and *L. salivarius* upregulated the expression of LCE1A, LCE1B, LCE1C, LCE1F, LCE2A, LCE2B, LCE2C, LCE2D, and LCE6A, which are constitutively expressed during healthy skin maturation (Figures 3B and S1). Notably, 10% *B. breve* led to increased LCE3 genes such as LCE3A, LCE3D, and LCE3E, which are typically upregulated in response to injury, inflammation, or barrier disruption, emphasizing their role in stress‐related or repair processes [22, 23]. The expression of FLG, a key component of the skin barrier, increased with 2.5% and 5% doses of *B. breve* and 10% *L. salivarius* . Additionally, *B. breve* , *L. reuteri* , and *L. salivarius* induced the expression of CASP14, an enzyme expressed predominantly in cornifying epithelia, which catalyzes the degradation of profilaggrin to FLG. The expression of IVL also increased in response to *B. breve* and *L. salivarius* . Furthermore, basal keratinocyte markers KRT5 and KRT14, along with differentiated keratinocyte markers KRT1 and KRT10, were upregulated at lower doses of *B. breve* . Notably, all doses of *B. breve* and 10% of *L. salivarius* induced the expression of KRT17, a keratinocyte activation marker associated with wound healing. Furthermore, cell‐type deconvolution analysis of the bulk RNA‐seq data reveals that 10% *B. breve* led to an increased fraction of proliferating keratinocytes (Figure S3A). Collectively, these findings illustrate that applying postbiotics from *B. breve* , *L. reuteri* and *L. salivarius* enhances key molecular signatures involved in epidermal barrier formation, reinforcing the structural framework of the skin epithelium.

### 2.4 Essential Keratinocyte Differentiation and Anti‐Inflammatory Genes in Skin Barrier Integrity

**FIGURE 4:**
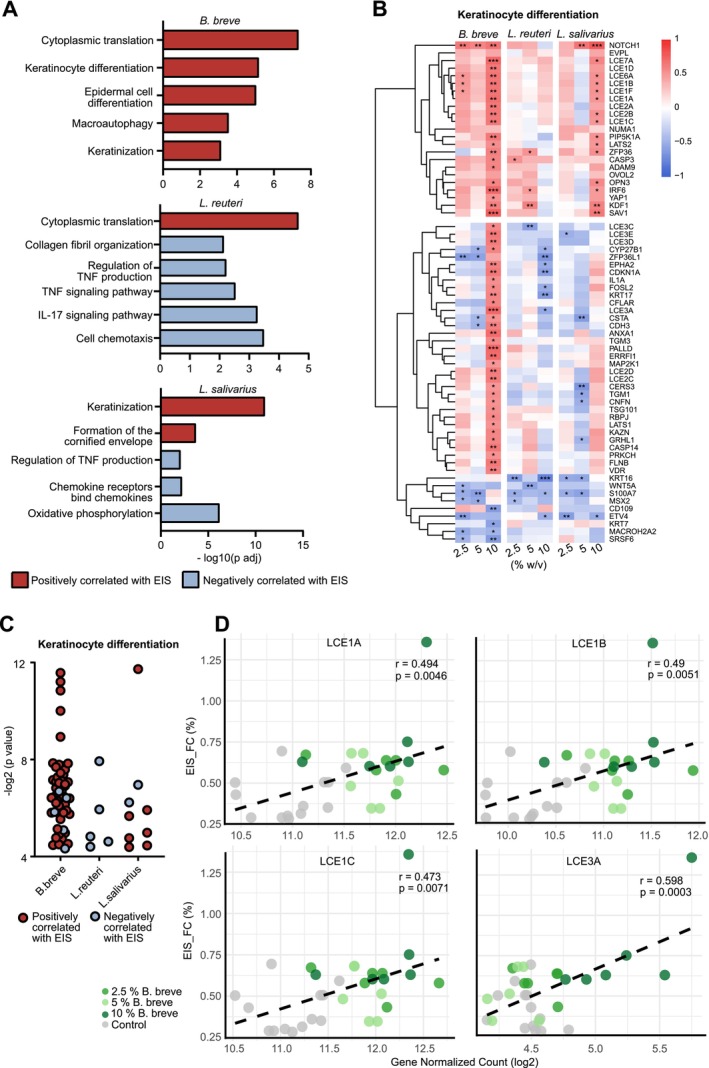
Keratinocyte differentiation positively correlated with increased skin epithelial barrier integrity (A) Strain‐specific GSEA pathway analysis of genes that correlate with EIS. (B) Heatmap of EIS‐correlated genes related to keratinocyte differentiation, the positive values (red) show positive correlation, whereas negative values (blue) show negative correlation. (C) *p*‐Value distribution of EIS correlated genes associated with keratinocyte differentiation. (D) Correlation plots of LCE1A, LCE1B, LCE1C, and LCE3A expression with EIS after *B. breve* treatment. EIS_FC, EIS fold change. *p < 0.05, **p < 0.01, ***p < 0.001.

An important objective of this study was to investigate the links between the skin barrier integrity and transcriptome changes. To achieve this, correlation analyses were performed to assess the relationship between each postbiotic's effect on skin epithelial barrier enhancement and its impact on transcriptomics. We found that the EIS values correlated significantly with 8059 *B. breve* , 2057 *L. reuteri* , and 3084 *L. salivarius‐*induced gene expression alteration in transcriptomic analysis. Gene set enrichment analysis (GSEA) of these gene sets revealed that the skin epithelial barrier integrity‐regulating effects of *B. breve* and *L. salivarius* were related to increased cytoplasmic translation and the keratinocyte differentiation pathways and linked biological processes, including epidermal differentiation, keratinization, and cornified envelope formation (Figure 4A). Furthermore, the correlation analysis revealed that *L. salivarius* regulates skin epithelial barrier integrity not just by upregulating keratinization but also through anti‐inflammatory mechanisms. However, we found that the effect of *L. reuteri* on skin epithelial barrier integrity was solely through the anti‐inflammatory pathways (Figure 4A). The key genes related to keratinocyte differentiation induced by *B. breve* , include LCEs, CASP14, NOTCH1, and KDF1, exhibited positive correlation with EIS values (Figure 4B,D). Notably, the number of positively correlated genes with EIS was highest for *B. breve* compared to the other two postbiotics, which shows its prominent regulatory role in keratinocyte differentiation compared to other postbiotics (Figure 4C). These findings indicate that skin epithelial barrier integrity, particularly, depends on keratinocyte differentiation and anti‐inflammatory pathways.

### 2.5 Innate Immune Response and Pro‐Inflammatory Pathways Regulation in the Skin by Postbiotics

**FIGURE 5:**
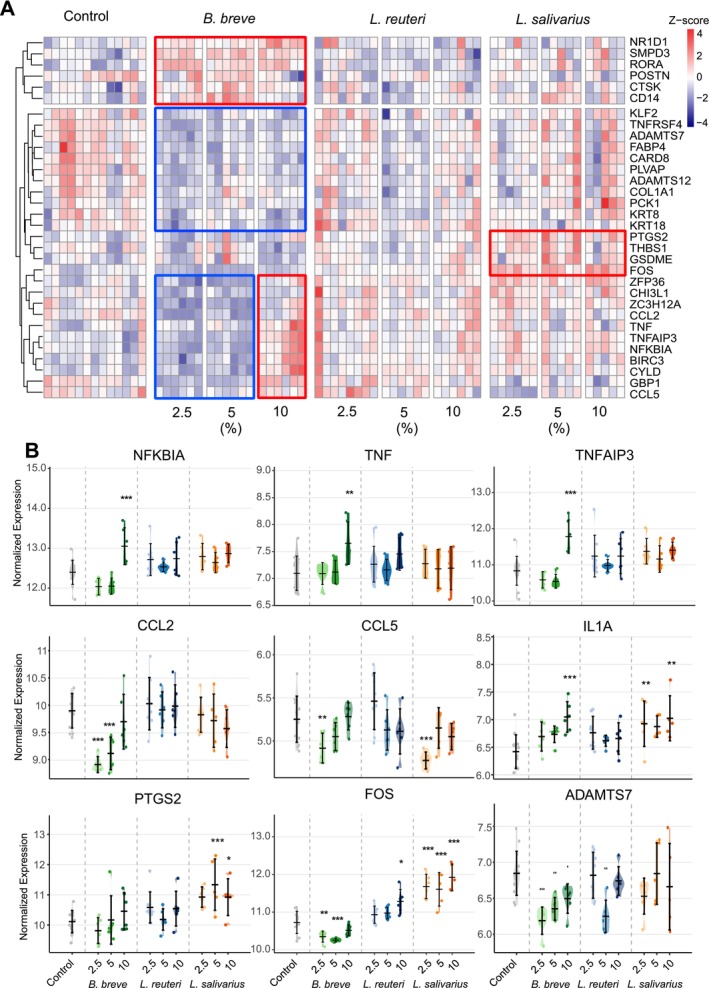
TNF response to postbiotics in ex vivo skin tissue. (A) The heatmap shows the significant genes related to response to tumor necrosis factor (GO:0034612) upon postbiotic treatment for 24 h. Not all the significant genes are shown. (B) The violin plots of significant genes related to tumor necrosis factor (GO:0034612). The data is shown as a normalized expression. *B. breve* , *Bifidobacterium breve* ; *L. reuteri* , *Limosilactobacillus reuteri*; *L. salivarius* , *Ligilactobacillus salivarius*. *p < 0.05, **p < 0.01, ***p < 0.001.

Postbiotic treatment modulated the innate immune response‐related pathways in a strain‐ and dose‐dependent manner as demonstrated in Figure 5A. Induction of an anti‐inflammatory effect in the skin is a key component of the treatment of many inflammatory skin diseases. The 2.5% and 5% doses of *B. breve* caused a significant downregulation of response pathways to tumor necrosis factor and multiple chemokines, as shown in Figure 5B. The expression levels of several key factors in the innate immune response, such as CCL2, CCL5, FABP4, FOS, TNFRSF4, ADAMTS7, ADAMTS12, and CARD8 were downregulated with low‐ and medium‐doses of *B. breve* treatment. These anti‐inflammatory effects of *B. breve* were unique and can be considered a positive influence on skin homeostasis. They were not observed with the other two postbiotics. In contrast, the high dose, 10%, of *B. breve* caused a significant increase in the markers of NF‐κB activation, such as TNFAIP3, NFKBIA and TNF. This is consistent with the upregulated response to the tumor necrosis factor pathway, as shown in the GSEA pathway analysis in Figure 2B. In addition, a key proinflammatory cytokine IL1A was increased with 10% *B. breve* treatment (Figure 5B).

Similarly, *L. salivarius* caused a dose‐dependent activation of innate immune response‐related pathways; however, without showing any apparent downregulation of these pathways in lower doses. Among its proinflammatory effects, FOS, a crucial protein in the formation of the AP‐1 transcription factor, was elevated across all tested doses of *L. salivarius* . Furthermore, the PTGS2 gene, which encodes the COX2 enzyme, has increased by 5% and 10% with *L. salivarius* treatment. 2.5% and 10% doses of *L. salivarius* caused upregulation of IL1A (Figure 5B). *L. reuteri* species did not cause any significant increase in innate immune response‐related genes, except for a slight increase in FOS protein.

Immune cell‐type focused deconvolution analysis of the bulk RNA‐seq data demonstrated that cytotoxic T cell and natural killer cell fractions decreased in *L. salivarius* (Figure S3B). Overall, these findings demonstrate that each postbiotic strain exhibited distinct and dose‐dependent effects on inflammation‐related genes in skin tissue. While low doses of *B. breve* displayed a notable anti‐inflammatory profile, higher doses of *B. breve* and *L. salivarius* exhibited proinflammatory tendencies, suggesting potential dose‐dependent immunomodulatory effects.

### 2.6 Protein Expressions Validate Biological Pathways Observed in Transcriptomics

**FIGURE 6:**
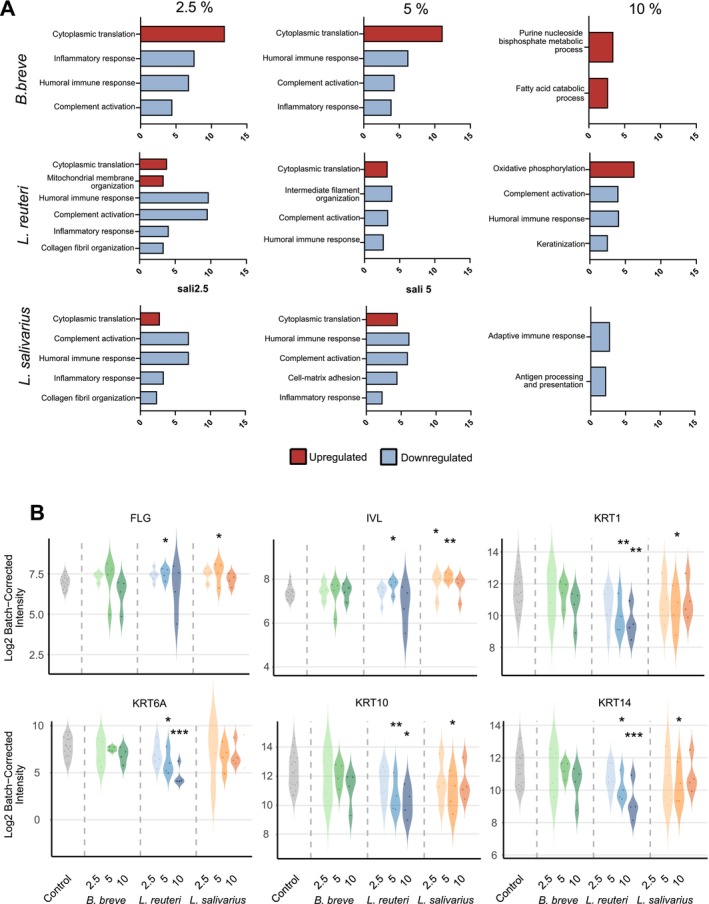
Untargeted proteomics analysis validated the transcriptomic results. (A) Bar plots of GSEA analysis of untargeted proteomics results. (B) Violin plots showing protein expression levels validated by targeted proteomics analysis for critical keratinocyte differentiation markers: filaggrin (FLG), involucrin (IVL), keratin 1 (KRT1), keratin 6A (KRT6A), keratin 10 (KRT10), and keratin 14 (KRT14). Protein expression data were shown as Log2 Batch−Corrected Intensity. *B. breve* , *Bifidobacterium breve* ; *L. reuteri* , *Limosilactobacillus reuteri*; *L. salivarius* , *Ligilactobacillus salivarius*. *p < 0.05, **p < 0.01, ***p < 0.001.

Proteomics analysis further validated these transcriptomic insights, identifying significant biological pathways modulated by the postbiotic treatments. GSEA revealed that the treatment with postbiotics led to significant enrichment of downregulated pathways including inflammatory response, complement activation, humoral immune response, cytoplasmic translation, fatty acid catabolic processes, purine nucleoside bisphosphate metabolic processes, collagen fibril organization, mitochondrial membrane organization, oxidative phosphorylation, antigen processing and presentation, adaptive immune response, intermediate filament organization, and cell‐matrix adhesion (Figure 6A). In addition, cytoplasmic translation, purine nucleoside bisphosphate metabolic process, fatty acid catabolic process, and oxidative phosphorylation pathways are upregulated. Violin plots demonstrated regulation of protein expression levels of critical keratinocyte differentiation markers such as FLG, IVL, KRT1, KRT6A, KRT10, and KRT14 upon postbiotic treatment (Figure 6B). The findings supported the anti‐inflammatory properties of postbiotics at lower doses.

### 2.7 Machine Learning Analysis Revealed Essential Genes Linked to Skin Barrier Integrity

**FIGURE 7:**
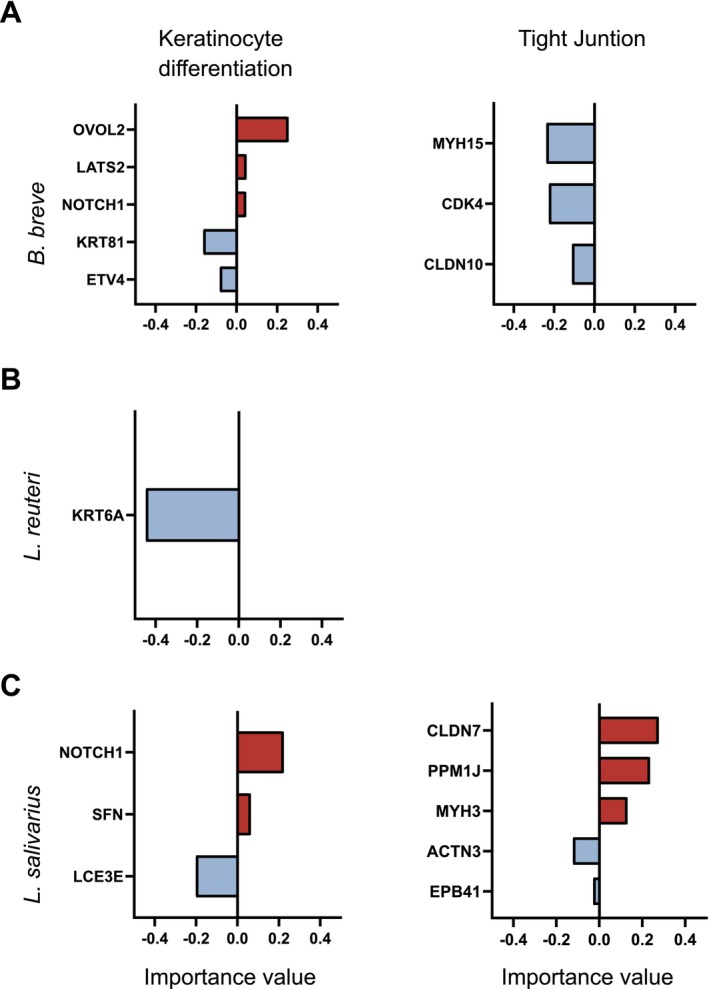
Genes involved in improving or disturbing skin barrier integrity by machine learning. Strain‐specific transcriptomic changes related to keratinocyte differentiation and tight junction correlated with EIS related to *B. breve* (A), *L. reuteri* (B), and *L. salivarius* (C) treatment. XGBoost machine learning was used to identify the best‐predicting genes involved in keratinocyte differentiation and tight junction correlation based on the fold changes of EIS data. *B. breve* , *Bifidobacterium breve* ; *L. reuteri* , *Limosilactobacillus reuteri*; *L. salivarius* , *Ligilactobacillus salivarius*.

We performed a machine learning analysis, namely the Extreme Gradient Boosting (XGBoost) regression model to deepen our understanding of the correlation between skin epithelial barrier integrity and transcriptomic changes related to keratinocyte differentiation and tight junctions in response to postbiotics. XGBoost is a robust, scalable, and efficient machine‐learning algorithm based on gradient boosting. It is widely used for supervised learning tasks such as classification and regression [26]. XGBoost analysis revealed key genes correlated with the regulation of skin epithelial barrier in a strain‐specific manner. Among these, the most critical genes associated with *B. breve* ‐induced skin barrier enhancement included OVOL2, KRT81, ETV4, LATS2, NOTCH1, MHY15, CDK4, and CLDN10 (Figure 7A). In contrast, KRT6A was found as one of the most important *L. reuteri‐*regulated genes linked to increased skin epithelial barrier integrity (Figure 7B). In addition, NOTCH1, SFN, CLDN7, PPM1J, MYH3, LCE3E, ACTN3, and EPB41 were the most important genes for *L. salivarius* ‐mediated skin epithelial barrier enhancement. These findings suggest a complex regulatory network involving keratinocyte differentiation and tight junction‐associated genes highlighting strain‐dependent mechanisms in postbiotic‐mediated skin barrier modulation.

### 2.8 NAM Regulates the Skin Epithelial Barrier Integrity Associated With Anti‐Inflammatory Functions

Transcriptomic analysis revealed that NAM treatment resulted in dose‐dependent downregulation of genes associated with keratinization in ex vivo human skin. The heatmap illustrates significant decreases in the expression of multiple keratinocyte differentiation‐related genes following treatment with NAM at concentrations of 1.5 and 6 mM compared to the untreated control (Figure S2A). Notably, key genes, including small proline‐rich protein and late cornified envelope family genes, are downregulated, particularly at 6 mM. These findings underscore the distinct inhibitory effect of NAM on keratinocyte differentiation pathways, highlighting its potential modulatory role in managing conditions associated with excessive keratinization.

Correlation analysis between transcriptomic changes and skin barrier integrity (measured as EIS fold‐change) following NAM treatment revealed distinct patterns. Negative correlations were observed for ADAM28 and C4BPA. Specifically, ADAM28, involved in lymphocyte adhesion and proteolytic remodeling, and C4BPA, a regulator of the complement system, may indicate detrimental inflammatory or tissue‐remodeling states. Conversely, positive correlations were found with indolamine 2,3 dioxygenase 1 (IDO1), a strong anti‐inflammatory gene converting tryptophan to kynurenine in the kynurenine pathway that has been shown to downregulate several immunologic mechanisms. In addition, KRT25 and DCTN5 are positively correlated and linked to structural integrity and intracellular transport in immune cells, respectively (Figure S2B). These findings suggest that NAM modulates skin gene expression by decreasing inflammatory mediators while simultaneously enhancing structural stability and immune tolerance, highlighting its protective potential for skin barrier integrity.

### 2.9 *B. breve* , *L. reuteri* , and NAM Healed the Skin Epithelial Barrier Damage Caused by Cocoyl Methyl Glucamide

**FIGURE 8:**
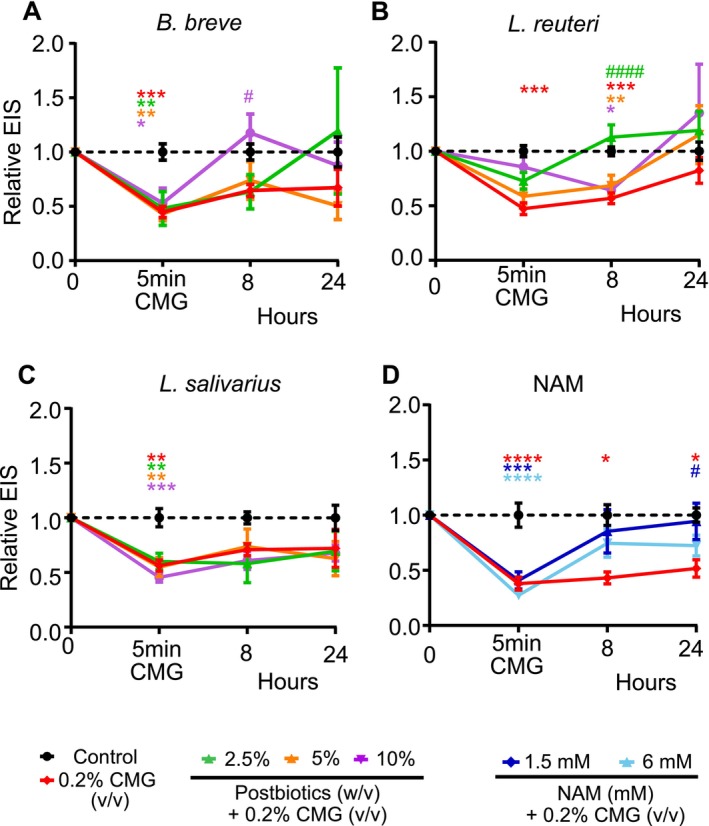
Postbiotics and NAM mitigate the skin epithelial barrier damage of surfactant (A) *B. breve* ; (B) *L. reuteri* ; (C) *L. salivarius* : (D) NAM. Postbiotics were applied to ex vivo skin tissues apically at doses of 2.5%, 5% and 10% (w/v), and EIS was measured at baseline, 8 and 24 h. For the experiments, at least two different donors with 2–3 technical replicates were used. In total, eight donors were used. *B. breve* , *Bifidobacterium breve* ; EIS, electrical impedance spectroscopy; *L. reuteri* , *Limosilactobacillus reuteri*; *L. salivarius* , *Ligilactobacillus salivarius*; NAM, nicotinamide. Statistical significance: *p* < 0.05, *p* < 0.01, *p* < 0.001, *p* < 0.0001 vs. control; #*p* < 0.05, ####*p* < 0.0001 vs. CMG.

To functionally assess the anti‐inflammatory and skin epithelial barrier‐enhancing effects of the postbiotics and NAM, we conducted an exposure and treatment experiment using ex vivo human skin. We applied a surfactant commonly used in detergents, cosmetics, personal care products, and shampoos, coco methyl glucamide (CMG), as a skin epithelial barrier‐disrupting agent. A 5‐min treatment of 0.2% CMG led to a rapid decline in skin epithelial barrier integrity, as evidenced by a decrease in EIS measurements. The treatment with *B. breve* , *L. reuteri* , and NAM of the native skins after the addition of CMG recovered the detrimental effect of CMG on the skin epithelial barrier (Figure 8). These data show the healing effect of certain postbiotics and NAM after skin epithelial barrier damage.

## 3 Discussion

The study investigated the effects of postbiotic preparations from *B. breve* , *L. reuteri* , *L. salivarius* , and NAM on skin barrier integrity, inflammation, and tissue responses in an ex vivo human skin model. We employed state‐of‐the‐art techniques, including EIS measurement for assessing skin epithelial barrier integrity [21, 27, 28], multiomics and machine learning analysis to reveal the molecular mechanisms of skin epithelial barrier regulation. We identified the key pathways and associated genes that regulate skin epithelial barrier integrity through postbiotics and NAM, including keratinocyte differentiation, formation of the cornified envelope, basal keratinocyte proliferation, cell junction proteins, innate immune response, and inflammation‐related pathways.

Lower concentrations (2.5% and 5%) of postbiotics of *B. breve* and *L. reuteri* significantly improved epithelial barrier integrity at 8 and 24 h post‐treatment, as shown by increased EIS values in both healthy and damaged skin models. In contrast, *L. salivarius* and NAM had a moderate impact on EIS. EIS applied to in vitro models is known to be a robust measure of the impact of topical substances on the skin barrier, and the study was further focused on molecular mechanisms of epithelial barrier regulation and its healing by postbiotics.

The postbiotic treatments upregulated crucial pathways related to skin barrier function, including keratinocyte differentiation, establishment of the skin barrier, intermediate filament organization, and retinoic acid metabolism, with distinct dose‐ and time‐dependent effects. Transcriptomic analysis of ex vivo skin tissues treated with postbiotic preparations to investigate the mechanisms underlying these barrier effects enabled examination of the influence of these agents on inflammatory pathways. At optimal doses such as 5% and 10%, *B. breve* and *L. reuteri* significantly increased skin barrier integrity by promoting keratinocyte differentiation. However, high doses of *B. breve* and *L. salivarius* triggered inflammatory pathways, including TNF and NF‐κB activation, suggesting a concentration threshold beyond which postbiotics may become pro‐inflammatory. *B. breve* showed additional beneficial effects, which were not exhibited by other postbiotics, namely the strong anti‐inflammatory effects in low doses.

It is important to note that postbiotic applications influenced keratinocyte differentiation at several checkpoints. *B. breve* increased the expression of genes associated with cornified envelope formation and keratinocyte differentiation (e.g., FLG, IVL, KRTs, LCEs, and CASP14) and modulated signaling cascades that support keratinocyte proliferation and differentiation. The increased expression of FLG, IVL, LCEs, and differentiated keratinocyte markers KRT1 and KRT10 genes indicates enhanced formation and stabilization of the keratinocyte cytoskeletal network, contributing to improved structural integrity and functionality of the epidermal barrier [3, 29]. At the same time, upregulated CASP14 enhanced FLG production through the cleavage of profilagrin [30]. In addition, the upregulated basal keratinocyte markers KRT5 and KRT14 show increased keratinocyte proliferation [31]. Increased expressions of LCE1B, LCE1C, LCE1F, LCE2A, LCE2B, LCE2C, LCE2D, and LCE6A show the effect of postbiotics on maintaining the healthy skin barrier properties, as these LCEs are constitutively expressed in healthy skin maturation [22]. However, increased keratinization can occur in both homeostatic and pathological contexts. The increased expression of LCE3 genes, including LCE3A, LCE3D, and LCE3E, associated with injury, inflammation, or barrier disruption, appears to be induced by excessive stimulation from bacterial products, particularly at high postbiotic doses [22, 23]. This excessive stimulation can be seen as upregulated pathways such as those involved in defense against and response to bacteria, particularly in high doses of postbiotics. These findings align with earlier research, which has indicated that inanimate formulations of lactic acid bacteria can enhance keratinocyte differentiation and tight junction function, culminating in increased skin epithelial barrier integrity [8].

We proceeded with correlating skin epithelial barrier integrity and transcriptomic analysis using both conventional correlation tests and a machine learning approach. The strain‐specific analysis of the correlation between the EIS fold change values and transcriptome revealed that *B. breve* enhances the skin epithelial barrier integrity through upregulated keratinocyte differentiation. However, the effect of *L. reuteri* and *L. salivarius* on skin epithelial barrier integrity was also dependent on their anti‐inflammatory properties, including downregulation of cytokine and chemokine production and signaling. These findings underscore the role of anti‐inflammatory mechanisms in supporting skin epithelial integrity. Yet, under basal conditions, such mechanisms alone appear insufficient to reinforce the barrier; rather, enhanced keratinization emerges as the more potent driver of improvement. In addition, the machine learning algorithm, XGBoost, revealed key genes related to keratinocyte differentiation and tight junction in the regulation of skin epithelial barrier integrity. These genes include transcription factors such as OVOL2, ETV4, NOTCH1, and TRIM16, structural proteins KRT6A, KRT81, and LCE3E, and key tight junction genes such as CLDN7, CLDN10, MYH3, and MYH15. It appears that postbiotics are fine‐tuning the balance between basal keratinocyte proliferation and keratinization through OVOL2, LATS2, ETV4, and NOTCH1 [32, 33, 34, 35]. These transcription factors are crucial for the timely differentiation of keratinocytes with maintaining keratinocyte integrity, preventing premature differentiation, and ensuring proper stratification [32, 33, 34, 35]. The regulators of keratinocyte proliferation via LATS2, CDK4, and SFN are also crucial for preventing defective and impaired stratification [35, 36]. Meanwhile, maintaining the structural integrity of keratinocytes with keratins and cell–cell adhesions is another important aspect that is regulated by postbiotics.

An intriguing aspect of our results is the dose‐dependent impact on pro‐inflammatory markers of *B. breve* , showing the activation of anti‐inflammatory genes in low doses and proinflammatory genes in high doses. Indeed, 2.5% *B. breve* had an anti‐inflammatory effect, marked by downregulation of innate immune system‐related pathways in transcriptomic analysis. In contrast, higher doses induced the upregulation of pro‐inflammatory genes. Obviously, high doses of these bacteria cause more extensive exposure to bacterial cell wall products, which can induce inflammation through pattern recognition receptors. Increased NFKBIA, TNFAIP3, TNF, PTGS2 and chemokines indicate activation of key inflammatory signaling cascades such as NF‐κB [37, 38]. These findings suggest a potential threshold beyond which stimulatory effects on immune activation overshadow beneficial anti‐inflammatory properties. Comparable patterns were seen with *L. salivarius* , where only elevated concentrations triggered the expression of AP‐1 factors, such as FOS and PTGS2. These concentration‐dependent responses underline the necessity of calibrating postbiotic formulations to maximize protective barrier enhancement while minimizing tissue stress or inflammation. In addition, postbiotics may influence metabolic reprogramming in keratinocytes, as evidenced by the upregulation of genes involved in oxidative stress responses and energy metabolism, ultimately strengthening the skin's structural network [39, 40, 41, 42].

This nuanced relationship between dose and outcome parallels the concept of hormesis [43], where moderate levels of specific bacterial components and/or metabolites can activate stress‐resistance pathways and barrier repair mechanisms. However, excessive amounts of the same postbiotics can overwhelm these systems and elicit inflammatory responses.

Deconvolution of bulk RNA‐seq data revealed additional, cell‐type–specific insights into the postbiotic effects on skin physiology. Treatment with *L. salivarius* resulted in a decrease in cytotoxic T cell and natural killer cell fractions, suggesting that this strain may suppress cell‐mediated immunity. This aligns with its immunomodulatory rather than regenerative effects. In contrast, treatment with 10% *B. breve* increased the fraction of proliferating keratinocytes, consistent with its transcriptomic signature of enhanced keratinocyte differentiation and cornified envelope formation. The expansion of proliferating keratinocytes likely reflects accelerated epidermal renewal, which contributes to the improved barrier integrity. Such strain‐ and dose‐dependent cell‐type shifts highlight the intricate balance between regeneration and inflammation in postbiotic‐driven epithelial regulation. Although *B. breve* exhibits clear anti‐inflammatory transcriptomic signatures at lower doses, it is important to note that substantial changes in immune‐cell fractions are unlikely to occur within 24 h. Further, non‐tissue‐resident population fractions, such as cytotoxic T cells, helper T cells, and regulatory T cells, are primarily determined by infiltration; therefore, our results reflect early transcriptional cues rather than actual population remodeling. Our findings in proteomics analysis supported our findings on transcriptomics. The GSEA analysis of untargeted proteomics results was parallel to the transcriptomics GSEA analysis. The anti‐inflammatory effects of postbiotics were prominent at lower doses, characterized by a decreased humoral immune response, complement system activation, and reduced inflammatory pathways. In addition, the downregulation of KRT6A, a gene highly related to *L. reuteri* ‐driven regulation of skin epithelial barrier integrity, as determined by our machine learning analysis, with the high concentration (10%) of *L. reuteri* , provides further evidence, as the 24‐h EIS of this condition is lower than its 8‐h time point. Further, downregulation of KRT1, KRT10, and KRT14 with higher *L. salivarius* concentration shows dysregulation of the keratinization process in these doses.

Our experiments comparing the mechanisms of action of NAM and postbiotics highlighted the distinct molecular landscape for skin barrier regulation. NAM is well known for its capacity to improve lipid synthesis, reduce transepidermal water loss, and inhibit inflammatory cytokines [17, 44, 45, 46]. In the present study, NAM displayed modest skin epithelial barrier improvement with dose‐dependent downregulation of keratinocyte differentiation. However, unlike postbiotics, NAM did not stimulate pro‐inflammatory genes, even at a high dose (6 mM). The correlation analysis of transcriptomics and EIS also supported the importance of its anti‐inflammatory capacities. The effect of NAM on skin epithelial barrier regulation was dependent on several key anti‐inflammatory genes and structural proteins. NAM upregulates IDO1, which catabolizes tryptophan, leading to rapid local tryptophan depletion that suppresses effector T cell activation and supports regulatory T cell development. Through the IDO1‐kynurenine and AhR pathways, IDO1 counteracts proinflammatory and tissue‐damaging cytokines [46]. These together counteract excessive proinflammatory response contributing to resolution and protection from tissue injury. Unlike postbiotics, NAM did not upregulate keratinization‐related genes, but it still enhanced epithelial integrity, indicating a distinct, yet complementary mode of action. These findings underscore the potential synergy between moderate concentrations of postbiotics and NAM, a concept that warrants further studies.

It should be noted that our study is not without limitations. We used an ex vivo skin model lacking a fully functional nervous system, lymphatic vessels, and blood circulation, therefore lacking the capacity for immune cell infiltration. Our study has a relatively low sample size, and all the donors are females. Future research should examine these interventions in more physiologically complete models or clinical settings. Moreover, the exact active fractions in each postbiotic preparation remain undefined, limiting fully detailed mechanistic insights. In the present study, whole cell preparations of heat‐killed bacteria were used. Accordingly, future investigations aiming to fractionate the exact molecular components in each postbiotic strain could identify which molecules promote keratinocyte differentiation while minimizing inflammatory mediators. Such insights would be vital for developing topically applied treatments or incorporating these postbiotic compounds into cosmeceuticals designed to optimize skin barrier health.

Collectively, our data highlight the distinct pathways in skin epithelial barrier regulation, with postbiotics and NAM exerting broad actions on skin biology. According to our data, the key pathways in maintaining and increasing the skin epithelial barrier are a healthy keratinocyte differentiation process, coupled with an anti‐inflammatory mechanism. In addition, machine learning analysis further revealed gene‐level regulators of barrier function, providing mechanistic insights for targeted interventions and research on skin epithelial barrier integrity biomarkers.

## 4 Materials and Methods

### 4.1 Postbiotics and Preparation

Postbiotic strains (Limosilactobacillus reuteri Skinbac SB02 [LMG P‐34068], *Bifidobacterium breve* Skinbac SB03 [LMG P‐34069], Ligilactobacillus salivarius Skinbac SB04 [LMG P‐34067]) were provided by Probiotical S.p.A, Novara, Italy. The bacterial strains were subjected to thermal inactivation at temperatures exceeding 75°C for 30–90 min. Following heat treatment, the strains were processed via spray‐drying and subsequently analyzed using flow cytometry with two fluorophores, TO and PI, to determine the total fluorescent units (TFU). This quantification enabled standardization to a final concentration of 10^9^ TFU per gram. The resulting powders were stored at 4°C for further analysis of their postbiotic properties.

### 4.2 Postbiotic and NAM Treatments on Ex Vivo Human Skin

Ex vivo human skin explants (NativeSkin) were purchased from Genoskin (Boston, MA, USA). These anonymized human skin samples were obtained from eight healthy female donors undergoing abdominoplasty procedures, all with written informed consent. None had a history of allergies or dermatologic disorders, and none used corticosteroids. The demographic information of the donors is listed in Table 1. Full ethical approval for the study was obtained from the French ethical research committee (Comité de Protection des Personnes), and authorization was granted by the French Ministry of Research. Each donor's skin was processed into stabilized 15 mm diameter round biopsies, which were provided in custom‐built plastic inserts for up to 7 days of viability. All postbiotics were diluted at 2.5%, 5%, and 10% (w/v) in PBS. 1.5 and 6 mM NAM were prepared in PBS as well. Ex vivo human skin samples were treated with 60 μL of diluted postbiotics and NAM and were applied on the surface of ex vivo human skin for a total of 24 h.

**TABLE 1:** Demographics of ex vivo skin tissue donors.

Donor #	Sex	Age	BMI	Fitzpatrick classification
1	F	54	23	2
2	F	52	45	2
3	F	40	23	3
4	F	56	28	2
5	F	41	27	2
6	F	46	23.9	2
7	F	47	25	3
8	F	46	28	3

### 4.3 EIS Measurements

EIS measurements were performed using Nevisense (SciBase, Sundbyberg, Sweden), like in the previous study (27, 28). EIS quantifies a material's opposition to alternating current at a range of frequencies, thus providing insights into tissue integrity. Before each measurement, we gently moistened the NativeSkin surface with PBS, removed residual fluid with sterile cotton gauze, and then placed the EIS probe on the surface. We recorded 35 frequency measurements (1 kHz to 2.5 MHz) at two depths in three permutations. Each sample was measured five times per time point to reduce technical variability. We measured baseline EIS values (0 h), applied the postbiotics or NAM, and then re‐measured EIS at 8 h. After the 8‐h measurement, we rinsed the tissue surface with PBS three times, re‐applied the treatments, and finally conducted the 24‐h EIS measurement. PBS‐treated skin served as a control at each time point, and we evaluated each condition in skin explants from multiple donors (*n* = 8). We averaged these values while statistically accounting for donor variability.

### 4.4 RNA Isolation

Following the 24‐h EIS measurement, we extracted each skin biopsy from the plastic inserts and immersed it in RNAlater (Thermo Fisher Scientific, Waltham, MA, USA). We homogenized each sample in 350 μL RLT buffer (Qiagen, Hilden, Germany) with tissue homogenizing beads in a Precellys 24 homogenizer. We isolated total RNA with the RNeasy Plus Micro kit (Qiagen, Hilden, Germany), quantified it with a NanoDrop 2000 Spectrophotometer (Thermo Fisher Scientific, Waltham, MA, USA), and assessed RNA integrity on a 2200 TapeStation (Agilent Technologies, Santa Clara, CA, USA). Only samples with an RNA integrity number (RIN) > 7.0 were used for downstream transcriptomic analyses.

### 4.5 RNA Sequencing Analysis

We performed RNA sequencing (RNA‐seq) using a high‐throughput next‐generation sequencing approach. The transcriptome sequencing was carried out on an Illumina NovaSeq X Plus instrument, with each run involving one 1.5B flow cell and a total of two flow cells. Single‐end reads of 100 base pairs were generated. Library preparation was conducted using the Illumina Stranded mRNA Prep Ligation protocol, allowing for a stranded workflow to preserve transcript orientation. Raw reads were first quality filtered and adapter‐trimmed with BBDuk (BBMap suite) under default parameters to remove low‐quality bases (Phred < 20) and residual adapter sequences. Cleaned reads were then aligned to the human reference genome (GRCh38.p14) using Subread (v2.0.7). Transcript features were annotated according to GENCODE Release 46, and gene‐level read counts were quantified using featureCounts (Subread package).

After obtaining raw read counts, batch effects related to individual donor variation were corrected with ComBat‐Seq in R (v4.2.2), using donor identity as a covariate. The batch‐corrected count matrix was then subjected to differential expression analysis with DESeq2 (v1.46). Normalization factors were internally calculated based on the geometric mean of read counts, and size factors were estimated accordingly. Genes with fewer than ten reads across all samples were filtered out to reduce noise. Differentially expressed genes (DEGs) were identified by applying an adjusted *p*‐value cutoff of 0.05 (Benjamini–Hochberg correction). For pathway and network analyses, we used the STRING database (2023) for functional enrichment. Differentially expressed genes (DEGs) were mapped to the *Homo sapiens* dataset using default settings. Enriched Gene Ontology biological processes with a false discovery rate (FDR) < 0.05 were considered statistically significant.

### 4.6 Correlation and Machine Learning Analysis

After filtering out genes with low average expression (mean count ≤ 10) and applying a log_2_ transformation, the remaining normalized gene counts were used as predictors for modeling EIS fold change at 24 h from baseline. For each gene, we performed univariate correlation analyses using the Pearson method to assess the linear relationship with the continuous EIS response. Correlations with a *p*‐value < 0.05 were considered statistically significant, providing an initial indication of the strength and direction of association between gene expression levels and barrier integrity.

We then implemented an XGBoost regression model using the caret package in R, with hyperparameters tuned via repeated cross‐validation. We focused on the genes that are part of keratinization and tight junction pathways. The dataset was split into training (70%) and test (30%) sets, with careful alignment of gene expression values and corresponding EIS measurements extracted from accompanying metadata to ensure data consistency and integrity. Specifically, the tuning grid explored multiple values for key parameters: the number of boosting iterations (nrounds: 100, 500, 1000, and 2500), maximum tree depth (max_depth: 1, 2, 3, and 5), and learning rate (eta: 0.001, 0.01, and 0.1), while gamma was held at 0 and minimum child weight (min_child_weight: 5, 10, and 20) was adjusted to manage tree complexity. A 5‐fold cross‐validation repeated ten times was used to evaluate model performance and optimize these parameters, ensuring a balanced trade‐off between bias and variance. Separate XGBoost models were constructed for each species combined with the control group to study potential species‐specific effects on EIS.

The initial comprehensive model was trained on the full set of genes from selected pathways over 10 iterations to assess its performance based on Pearson correlation and root mean square error (RMSE) between predicted and observed EIS fold change values. To enhance model interpretability, a parsimonious model was subsequently constructed using the top 50 genes identified by feature importance metrics, thereby focusing on the most influential predictors for downstream biological validation.

### 4.7 Bulk RNA‐Deconvolution Analysis

Cell‐type deconvolution was performed with DISSECT as the base package, using a simulation‐calibrated workflow and two fully independent reference tracks analyzed end‐to‐end without mixing [47]. Specifically, an immune cell‐focused analysis was performed using scRNA data from Reynolds et al. [48], and a keratinocyte subtype‐focused analysis was performed using the dataset from Scholaert et al. [49]. Simulations used 10,000 pseudo‐bulk samples with 100,000 cells per sample, 50% sparsity (dropout emulation), Dirichlet‐like mixing over *α* ∈ [0.1, 0.9], and per‐batch log1p–MinMax normalization; both simulated and test matrices were normalized to counts per million, duplicates and low‐variance genes were removed at var_cutoff = 0.01. DISSECT's fraction estimator was instantiated as an ensemble of feed‐forward MLPs with six hidden layers (2048, 1024, 512, 256, 128, 64), ReLU hidden activations, softmax output to enforce non‐negativity and unit‐sum constraints, KL‐divergence loss with consistency regularization, learning rate = 1 × 10–5, batch size = 2048, 10,000 optimization steps, and no dropout. We applied DISSECT's recommended logit‐to‐SRM reconciliation to promote sparse, simplex‐constrained solutions and averaged predictions across ten independently initialized models. Proportions were estimated on the probability simplex within each analysis. Table S1 lists the genes that are significant in our dataset and overlap with those in the single‐cell datasets.

All inference was performed on uncentered percentages with a prespecified modeling hierarchy applied independently to each cell type. The linear mixed‐effects model (Percent ~ Condition + (1|Donor)) was used to account for between‐donor baselines. From the fitted model we obtained estimated marginal means and conducted Dunnett‐style contrasts versus control samples; within deconvolution analysis, raw *p*‐values from all contrasts were adjusted by Benjamini–Hochberg to control the false discovery rate.

### 4.8 Proteomic Sample Preparation and LC–MS/MS Analysis

Samples were received as 50 μL RIPA lysates (0.5 μg μL^−1^ total protein). Proteins were reduced and alkylated with 10 mM tris(2‐carboxyethyl)phosphine (TCEP)/40 mM 2‐chloroacetamide (CAA), followed by incubation for 5 min at 95°C. Plates were then cooled for 10 min at room temperature. Subsequent processing was carried out using an automated SP3 (autoSP3) workflow Müller et al. [50] adapted for the Opentrons Flex liquid‐handling workstation. To each well, 7 μL of magnetic bead suspension (50 μg μL^−1^)—a 1:1 mixture of Sera‐Mag SpeedBeads Carboxyl Magnetic Beads, hydrophilic (Cytiva 45152105050250) and hydrophobic (Cytiva 65152105050250)—was added, followed by 195 μL of 100% ethanol (final concentration ≈75%). Samples were subjected to repetitive shaking (1500 rpm, 30 s; 400 rpm, 90 s; nine cycles, 18 min total) to promote protein binding. Beads were washed sequentially with 80% ethanol (2 × 180 μL, 1500 rpm, 90 s) and 100% acetonitrile (180 μL, 1500 rpm, 90 s), and proteins were finally resuspended in 120 μL of 100 mM ammonium bicarbonate (ABC) buffer. Samples were sonicated for 5 min at 37 kHz, then digested by adding 10 μL of 0.1 μg μL^−1^ Trypsin/Lys‐C. Plates were incubated at 37°C for 17–18 h. Digestion was quenched with 15 μL of 10% formic acid (FA) to a final concentration of 1% FA. Plates were centrifuged at 400× *g* for 2 min and mixed for 5 min at 1500 rpm. The supernatant (magnetic rack) was transferred to a 96‐well collection plate. Peptides were dried under vacuum (SpeedVac, Thermo Fisher SPD120) and reconstituted in 3% acetonitrile/0.1% FA. Peptide concentrations were determined using the Pierce Colorimetric Peptide Assay Kit, and 200 ng of each digest was loaded onto Evotips (Evotip Pure) following the manufacturer's protocol. Evotips were rinsed with 0.1% formic acid in acetonitrile, activated with isopropanol, equilibrated with 0.1% formic acid, and loaded with 20 μL of 10 ng μL^−1^ peptide solution. After two washes with 0.1% formic acid, Evotips were stored at 4°C and inserted into the Evosep One autosampler immediately prior to analysis.

Liquid chromatography was performed on an Evosep One system operating under the 60 samples‐per‐day (60SPD) preset method. Peptides were separated on an Evosep C18 column (8 cm × 150 μm, 1.5 μm particle size; Performance EV1109) maintained at 40°C. Mass spectrometric analysis was carried out on a ZenoTOF 7600 mass spectrometer (SCIEX, Framingham, MA, USA) operating in positive ion mode with data‐independent acquisition (DIA) using the Scanning SWATH approach. Ionization was performed at 4500 V with a source temperature of 100°C. Source gas settings were: gas 1 = 25 psi, gas 2 = 15 psi, curtain gas = 45 psi, and collision gas = 7 psi. The MS1 scan range was m/z 400–1500 with a declustering potential of 80 V. Data‐independent acquisition (DIA) employed 65 variable isolation windows spanning m/z 399.2–996.8, with a 15 ms accumulation time per window and collision energies of 21–48 V. The TOF‐MS/MS scan range was m/z 230–1400. Unit resolution was applied at Q1 with a fixed charge state of 2, and the Zeno trap was enabled to enhance MS/MS sensitivity.

Raw data were processed using Spectronaut v19 (Biognosys AG, Switzerland) in directDIA+ mode with default parameters. The search database consisted of the *Homo sapiens* reference proteome (UniProt UP000005640; downloaded 7 September 2022). Protein groups were identified and quantified using Spectronaut's integrated FDR control. Processed quantitative data were exported for downstream statistical and bioinformatic analysis in R. Only proteotypic peptides were retained for analysis. Protein intensities were log‐transformed and differential protein abundance between each experimental condition and the control group was determined using two‐sided Student's *t*‐tests (implemented via compare_means() in *ggpubr*) with Benjamini–Hochberg correction for multiple testing. The control condition was set as “control,” and only proteins with ≥ 3 replicates per group were included.

### 4.9 Data Analysis and Statistics

All statistical analyses were conducted using R software (version 4.2.2) and GraphPad Prism (version 9.0). For EIS measurements, one‐way ANOVA was performed, followed by post hoc Tukey's tests to account for multiple comparisons.

## Funding

This study was funded by a grant from Swiss National Science Foundation (Bern), Stanford University, Leading House for the Latin American Region (Seed Money Grant), European Union (EU CURE, EU Syn‐Air‐G), Novartis Research Institutes (Basel, Switzerland), Stanford University (Redwood City, Calif), Seed Health (Boston, USA) and SciBase (Stockholm, Sweden), CSC scholarship program of China (No. 202008210164).

## Conflicts of Interest

M.A. has received research grants from the Swiss National Science Foundation, Bern; research grant from Stanford University; Leading House for the Latin American Region, Seed Money Grant. She is the Scientific Advisory Board member of Stanford University Sean Parker Asthma Allergy Center, CA; Advisory Board member of LEO Foundation Skin Immunology Research Center, Copenhagen; and Scientific Co‐Chair of World Allergy Congress (WAC) Istanbul, 2022, Scientific Programme Committee Chair, EAACI. C.A.A. has received research grants from the Swiss National Science Foundation, European Union (EU CURE, EU Syn‐Air‐G), Novartis Research Institutes (Basel, Switzerland), Stanford University (Redwood City, Calif), Seed Health (Boston, USA) and SciBase (Stockholm, Sweden); is the Co‐Chair for EAACI Guidelines on Environmental Science in Allergic diseases and Asthma; is on the Advisory Boards of Sanofi/Regeneron (Bern, Switzerland, New York, USA), Stanford University Sean Parker Asthma Allergy Center (CA, USA), Novartis (Basel, Switzerland), Glaxo Smith Kline (Zurich, Switzerland), Bristol‐Myers Squibb (New York, USA), Seed Health (Boston, USA), and SciBase (Stockholm, Sweden); and is the Editor‐in‐Chief of Allergy. I.O. is chair of the EAACI Epithelial Cell Biology Working Group. R.D. is a non‐executive Board Director at Seed Health Inc. Y.P., D.Y., H.B., S.A., X.B., P.W., A.G.‐S., M.L., O.A., C.Z. declare no relevant conflicts of interest. T.J. is a current employee of Seed Health, A.A. is a consultant to Seed Health, and R.D., S.S., and C.A. are former employees of Seed Health.

## Supporting information


**Figure S1:** Regulation of keratinocyte differentiation genes with postbiotics. The violin plots of significant genes related to keratinocyte differentiation (GO:0030216). The data is shown as normalized expression values. *B. breve: Bifidobacterium breve*; *L. reuteri: Limosilactobacillus reuteri*; *L. salivarius: Ligilactobacillus salivarius*.
**Figure S2:** NAM‐induced transcriptional changes in keratinization and their correlation with EIS. (A) Heatmap illustrating genes in the keratinization pathway. (B) Correlation plots of ADAM28, C4BPA, KRT25, DCTN5, and IDO1 expression with EIS after NAM treatment.

**Figure S3:** Strain‐specific cell type fraction changes related to keratinocyte subtypes (A), and immune cells (B). *B. breve: Bifidobacterium breve*; *L. reuteri: Limosilactobacillus reuteri*; *L. salivarius: Ligilactobacillus salivarius*.

**Table S1:** Genes significantly identified in our dataset that overlap with single‐cell transcriptomic datasets.

## Data Availability

The data that support the findings of this study are available from the corresponding author upon reasonable request.
