# Supplementary material for: Postbiotics and Nicotinamide Utilize Distinct Mechanisms to Improve Skin Barrier Integrity, Inflammation, and Keratinocyte Differentiation
Source: Allergy. 2026 Jan 23;81(3):830–47. doi: 10.1111/all.70225 (PMC12954566; doi:10.1111/all.70225)
Supplement: Supplementary file 1 — Figure S1: Regulation of keratinocyte differentiation genes with postbiotics. The violin plots of significant genes related to keratinocyte differentiation (GO:0030216). The data is shown as normalized expression values. B. breve: Bifidobacterium breve ; L. reuteri: Limosilactobacillus reuteri; L. salivarius: Ligilactobacillus salivarius. Figure S2: NAM‐induced transcriptional changes in keratinization and their correlation with EIS. (A) Heatmap illustrating genes in the keratinization pathway. (B) Correlation plots of ADAM28, C4BPA, KRT25, DCTN5, and IDO1 expression with EIS after NAM treatment. [file ALL-81-830-s001.pdf]

Postbiotics and Nicotinamide Utilize Distinct Mechanisms to Improve Skin Barrier Integrity, Inflammation, and Keratinocyte Differentiation

Short title: The Effects of Postbiotics and NAM on Skin

Yagiz Pat[1*], Duygu Yazici[1*], Huseyn Babayev[1*], Sena Ardicli[1,2], Xiangting Bu[1], Sheri Simmons[3], Anthony Almada[3], Christine Avena[3], Tye Jensen[3], Raja Dhir[1], Patrick Westermann[1,4], Asuncion Garcia-Sanchez[1,5], Manru Li[1], Ozge Ardicli[1,6], Can Zeyneloglu[1], Marco Pane[7], Angela Amoruso[7], Christoph Messner[4], Ismail Ogulur[1], Yasutaka Mitamura[1], Mubeccel Akdis[1], Cezmi A. Akdis[1]

*These authors contributed equally to this work.

[1]Swiss Institute of Allergy and Asthma Research (SIAF), University of Zurich, Davos, Switzerland

[2]Department of Genetics, Faculty of Veterinary Medicine, Bursa Uludag University, Bursa, Türkiye

[3]SEED Inc. Co., Los Angeles, California, USA

[4]Precision Proteomics Center, Davos, Switzerland

[5]Department of Biomedical and Diagnostic Sciences, University of Salamanca, Salamanca, Spain

[6]Division of Food Processing, Milk and Dairy Products Technology Program, Karacabey Vocational School, Bursa Uludag University, Bursa, Türkiye

[7]Probiotical Research S.r.l., Novara, Italy

Corresponding author:

Cezmi A. Akdis, Swiss Institute of Allergy and Asthma Research (SIAF), University of Zurich, Herman-Burchard-Strasse 9, CH-7265 Davos Wolfgang, Switzerland, e-mail: cezmi.akdis@siaf.uzh.ch

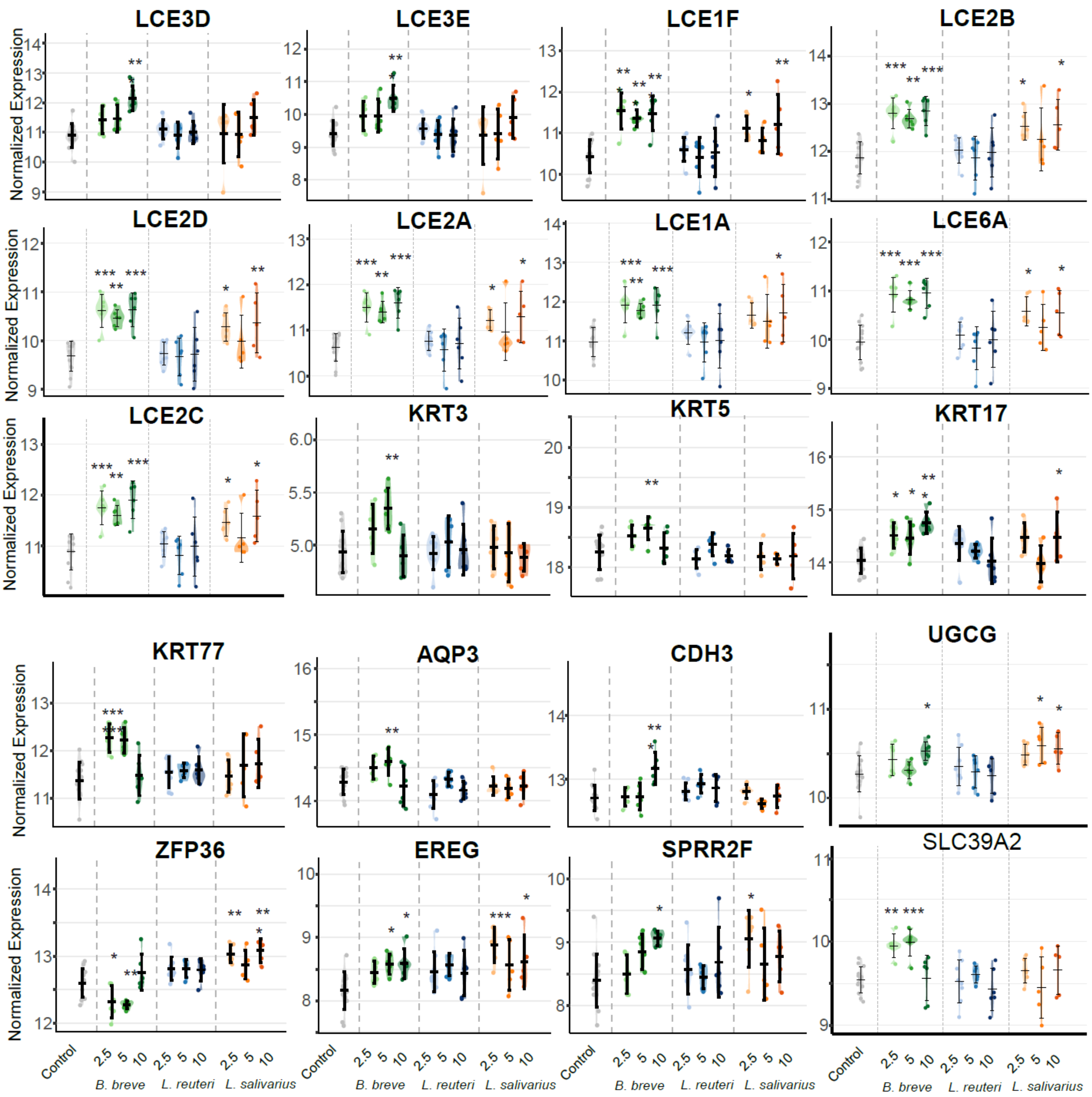

Supplementary Figure 1. Regulation of keratinocyte differentiation genes with postbiotics. The violin plots of significant genes related to keratinocyte differentiation (GO:0030216). The data is shown as normalized expression values. *B. breve: Bifidobacterium breve; L. reuteri: Limosilactobacillus reuteri; L. salivarius: Ligilactobacillus salivarius.*

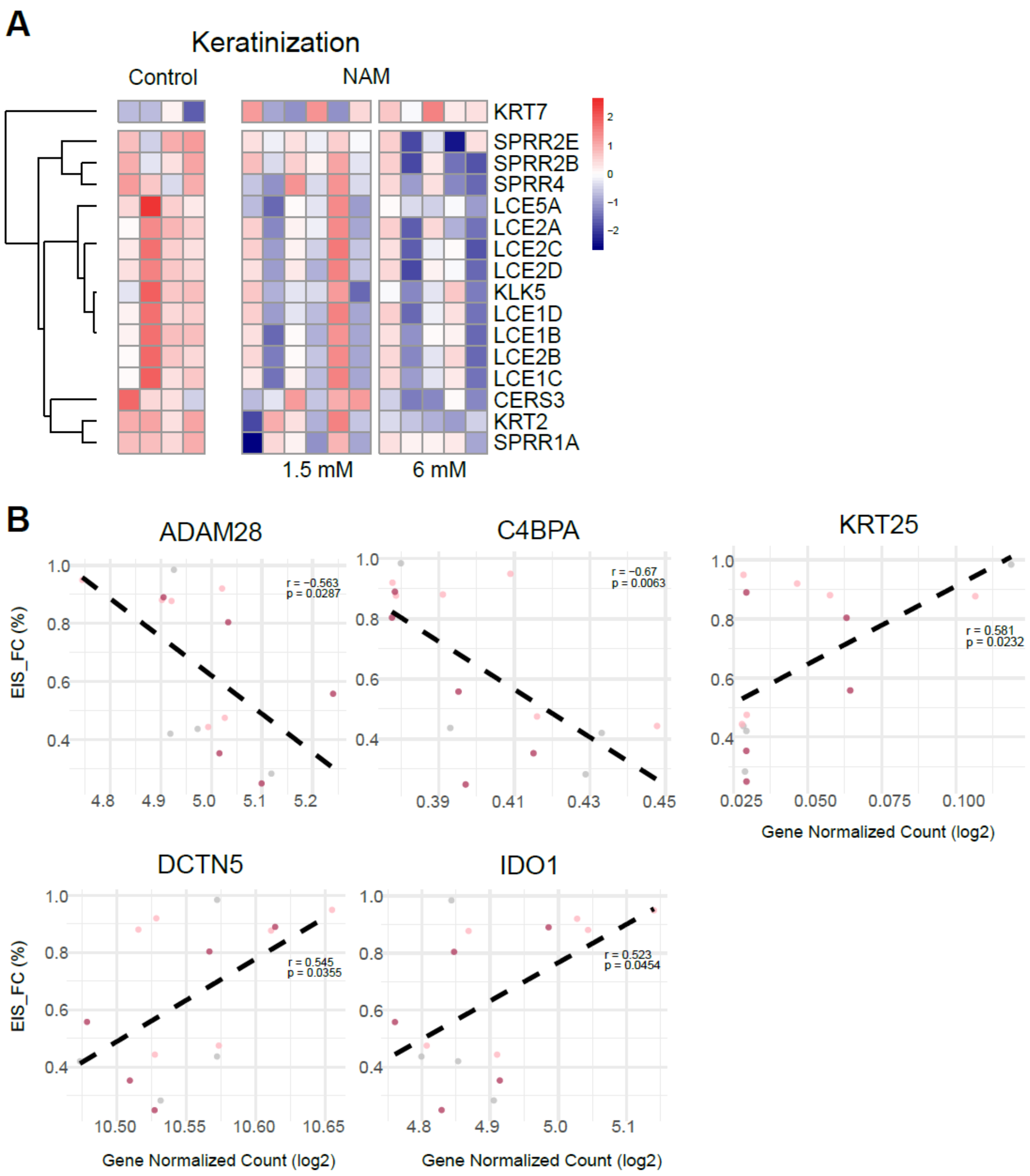

Supplementary Figure 2. NAM-induced transcriptional changes in keratinization and their correlation with EIS. (A) Heatmap illustrating genes in the keratinization pathway. **(B)** Correlation plots of ADAM28, C4BPA, KRT25, DCTN5, and IDO1 expression with EIS after NAM treatment.
