# Supplementary figures and images for: Postbiotics and Nicotinamide Utilize Distinct Mechanisms to Improve Skin Barrier Integrity, Inflammation, and Keratinocyte Differentiation
Source: Allergy. 2026 Jan 23;81(3):830–47. doi: 10.1111/all.70225 (PMC12954566; doi:10.1111/all.70225)

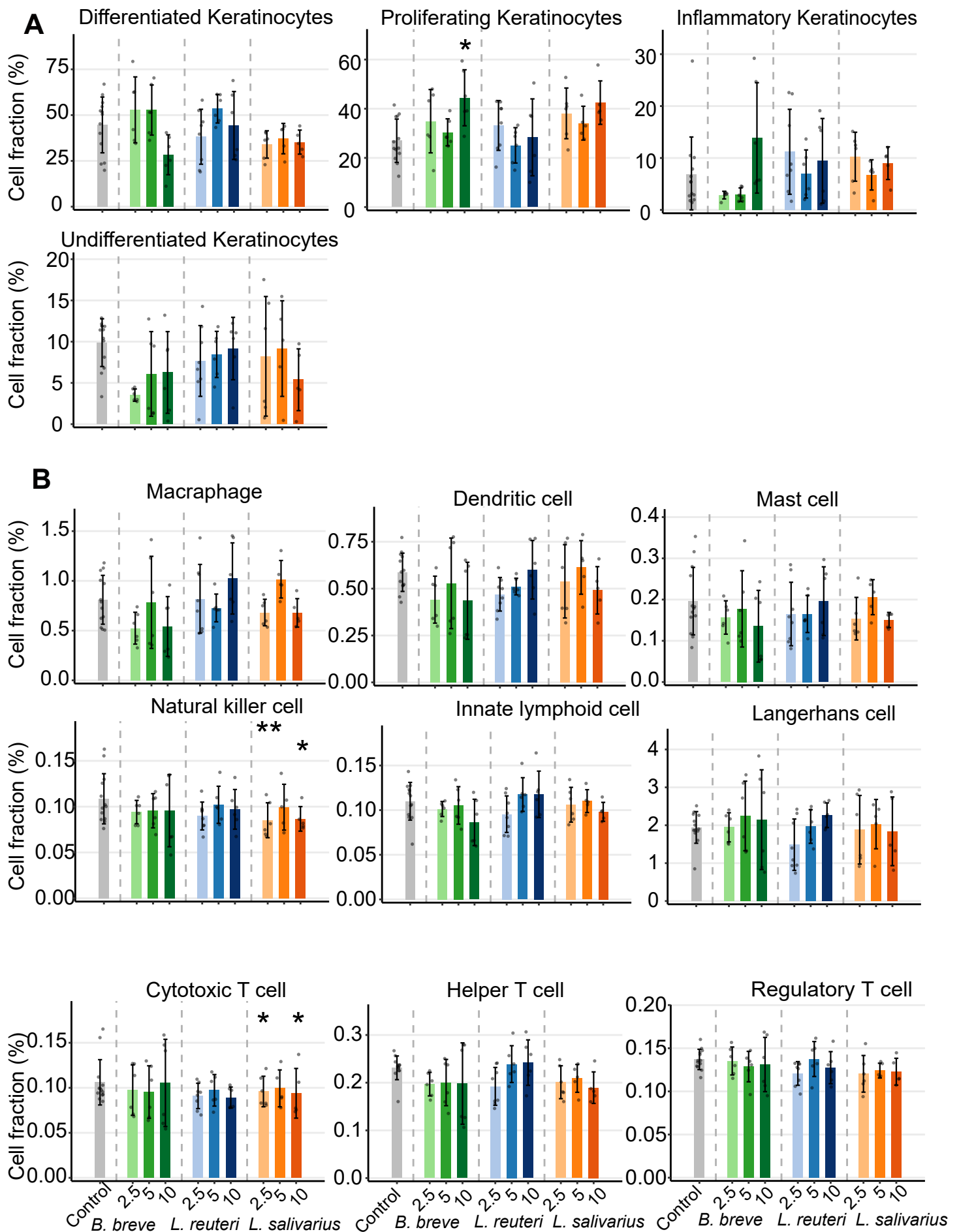

Supplement: Supplementary file 2 — Figure S3: Strain‐specific cell type fraction changes related to keratinocyte subtypes (A), and immune cells (B). B. breve: Bifidobacterium breve ; L. reuteri: Limosilactobacillus reuteri; L. salivarius: Ligilactobacillus salivarius. [file ALL-81-830-s003.pdf]
